# Bone Mineral Density Trends During the First Year After Laparoscopic Sleeve Gastrectomy—a Cohort Study on 241 Patients

**DOI:** 10.1007/s11695-021-05661-x

**Published:** 2021-08-27

**Authors:** Elisabeta Malinici, Anca Sirbu, Miruna Popa, Marian Andrei, Sorin Ioacara, Catalin Copaescu, Simona Fica

**Affiliations:** 1 Department of Endocrinology, Carol Davila University of Medicine and Pharmacy, Dionisie Lupu Street, no. 37, Sector 2, 020021 Bucharest, Romania; 2 Elias University Emergency Hospital, Marasti bd, no 17, Sector 1, 011461 Bucharest, Romania; 3 General Surgery Department, Ponderas Hospital, 85A Nicolae G Caramfil Street, Sector 1 , 014142 Bucharest, Romania

**Keywords:** Obesity, Sleeve gastrectomy, Bone mineral density

## Abstract

**Purpose:**

Laparoscopic sleeve gastrectomy (LSG) is an effective weight loss procedure, but detrimental effects on bone health have been described. We aimed to assess the dynamics of regional and total bone mineral density (BMD) in a cohort of patients undergoing LSG and to capture gender differences in terms of evolution.

**Materials and Methods:**

We conducted a retrospective study on 241 patients who underwent LSG to determine the regional and total BMD changes at 6 and 12 months after the intervention.

**Results:**

One hundred ten males and 140 females (97 pre-, 43 postmenopausal) were included. Mean baseline body mass index (BMI) was 44.16 ± 6.11 kg/m^2^ in males and 41.60 ± 5.54 kg/m^2^ in females, reaching 28.62 ± 4.26 kg/m^2^ and 27.39 ± 4.2 kg/m^2^, respectively, at 12 months. BMD showed a continuous decline, with significant loss from 6 months postoperatively. There was a positive correlation between BMD and BMI decline at 12 months (*r* = 0.134, *p* < 0.05). Total BMD loss at 12 months was significantly greater in males than premenopausal females, independent of BMI variation and age. During the first 6 months, men lost significantly more bone mass than premenopausal and postmenopausal women (BMD variation was 2.62%, 0.27%, 1.58%, respectively). The second period (6–12 months) was similar in all three groups, revealing a further steady (~ 1.4%) BMD decline.

**Conclusions:**

Our results are consistent with previous findings that LSG negatively impacts BMD, stressing the importance of bone health-oriented measures in postoperative care. Moreover, the impact that seems more significant in males warrants future exploration, as it might change clinical practice.

**Graphical abstract:**

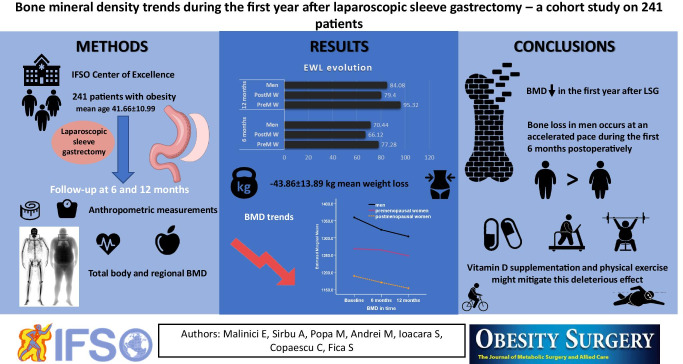

## Introduction

Obesity is a major public health issue, with a global rise in prevalence that has reached pandemic proportions over the past decades [1, 2]. Paralleling these trends and aided by the evolution of laparoscopic techniques, bariatric surgery has also shown a significant expansion. The most commonly performed interventions are Roux-en-Y gastric bypass (RYGB) and laparoscopic sleeve gastrectomy (LSG), with the latter becoming increasingly popular in the past years [3, 4]. LSG has proven clear beneficial effects on weight loss (both short-term and sustained), as well as improvement of obesity-related morbidity and mortality [5–9]. However, a matter of concern is the impact of bariatric surgery on bone homeostasis [10, 11]. Altered bone metabolism, accelerated bone loss, and increased fracture risk have been widely reported in patients undergoing RYGB [12–14], while data on the impact of LSG on bone health are limited [15, 16].

Dual-energy X-ray absorptiometry (DXA) scans are the most commonly performed investigations in clinical practice for bone mass assessment. Bone mineral density (BMD) has been long shown to correlate with fracture risk [17]. It is useful both as a diagnostic tool and for monitoring changes associated with either therapy or other detrimental factors [18, 19].

In this study, we aimed to assess the dynamics of regional and total BMD in a cohort of patients with obesity undergoing LSG, 6 and 12 months after the intervention, and to capture possible gender differences in terms of evolution.

## Materials and Methods

### Study Population

We conducted a retrospective study using prospectively collected data on a cohort of adult patients with obesity who underwent LSG at an International Federation for the Surgery of Obesity and Metabolic Disorders (IFSO) accredited Center of Excellence in Romania. The study was conducted with approval of the local Ethics Committee and following the ethical standards of the Helsinki Committee for Human Rights. Written informed consent was obtained from all patients.

Inclusion criteria were in accordance with guideline recommendations for bariatric surgery [20], while exclusion criteria consisted of age under 18 years old, use of medication impacting bone metabolism (other than calcium, vitamin D or hormonal contraception), known metabolic bone disease, and previous bariatric procedures. Three hundred forty patients met the inclusion criteria. Of them, 308 had complete baseline evaluation. Fifty-seven patients missed the 6 months follow-up visit, while another 29 missed the 12-month evaluation. The final analysis was performed on the 241 patients that had complete baseline and postoperative follow-up evaluation. All patients were provided professional nutritional and lifestyle recommendations, as advised by available guidelines at the time of intervention [20].

### Anthropometric Measurements

At every visit, body weight, height, waist and hip circumference were measured. Body mass index (BMI) was calculated as weight (in kilograms) divided by the square of height. Excess weight loss percentage (EWL) was calculated as [(Preoperative BMI − current BMI)/(preoperative BMI − 25)] × 100.

### Scan Procedure—Dual-Energy X-ray Absorptiometry (DXA)

DXA scans were performed using Lunar iDXA Forma (GE Healthcare). Total body and regional values of BMD (g/cm^2^) were recorded. All scans were performed by an ISCD-certified DXA technologist and analyzed with the same software.

### Statistical Analysis

Statistical analysis was performed using SPSS software, version 23.0. Quantitative variables were expressed as the mean ± standard deviation (SD). Continuous variables were compared using Student *t* test. Student paired *t* test was used to analyze the statistical significance of parameter variations during follow-up. All analyses were 2-tailed, with *p* < 0.05 considered statistically significant. Repeated Measures ANOVA test was used to assess time and gender effect for patients undergoing LSG. Effects were measured using Wilk’s Lambda test using a significance level of 0.001. Univariate general linear model (GLM) has been used to determine the relationship between BMD variation after 12 months and gender (including menopausal status for women), adding %BMI and age as covariates. Bonferroni post hoc test was applied for multiple comparisons.

## Results

A total of 241 subjects (140 women (58.1%) and 101 men (41.9%)) were included. Age ranged from 19 to 67 years, with a mean of 41.66 ± 10.99 and no significant difference between genders. Mean baseline BMI was 44.16 ± 6.11 kg/m^2^ in males and 41.60 ± 5.54 kg/m^2^ in females, decreasing to 28.62 ± 4.26 kg/m^2^ and 27.39 ± 4.2 kg/m^2^, respectively, 12 months postoperatively. The evolution of anthropometric parameters is presented in Table 1.

**Table 1:** Anthropometric characteristics at baseline, 6 and 12 months of follow-up, grouped by gender

	Baseline		6 months		12 months		p^2^	
	Males	Females	Males	Females	Males	Females	Time effect	Time x gender effect
	(*n* = 101)	(*n* = 140)	(*n* = 101)	(*n* = 140)	(*n* = 101)	(*n* = 140)		
BMI (kg/m^2^)	44.16 ± 6.11	41.6 ± 5.54	31.32 ± 4.69	30.15 ± 4.5	28.62 ± 4.26	27.39 ± 4.2	<.001	<.001
Weight (kg)	142.94 ± 21.32	114.71 ± 15.49	101.38 ± 16.23	83.11 ± 12.44	92.59 ± 14.38	75.52 ± 11.86	<.001	<.001
WHR	1.06 ± 0.08	0.95 ± 0.08	1.01 ± 0.07	0.92 ± 0.08	0.97 ± 0.08	0.91 ± 0.08	<.001	<.001
WC (cm)	139.82 ± 14.67	123.37 ± 12.92	110.84 ± 12.14	102.1 ± 11.98	102.97 ± 11.93	95.28 ± 11.17	<.001	<.001
HC (cm)	132.32 ± 13.12	130.16 ± 11.05	110.17 ± 10.09	110.33 ± 9.7	105.67 ± 8.62	104.25 ± 7.97	<.001	NS

^1^*BMI* body mass index, *WHR* waist-hip ratio, *WC* waist circumference, *HC* hip circumference

^2^Derived by using repeated measures ANOVA

^3^Values were expressed by Mean ± SD

BMD decreased at almost all analyzed sites at 6 months post-LSG and continued to decline during the subsequent 6 months. Total BMD followed the same downward trend (1.220g/cm^2^ at 12 months vs 1.292g/cm^2^ at baseline, *p*<0.001). An exception was leg BMD, which showed no decline at 6 months but did decrease at 12 months postoperatively (1.328 g/cm^2^ vs 1.344g/cm^2^, *p*<0.001). BMD and BMI decline correlated positively at 12 months (*r*=0.134, *p*<0.05). BMD decline and EWL showed no correlation. Results are summarized in Table 2.

**Table 2:** Densitometric variables at baseline, 6 and 12 months of follow-up

	Baseline	6 months	12 months
Head BMD (g/cm^2^)	2.201 ± 0.28	2.190 ± 0.27	2.185 ± 0.27^*b^
Arms BMD (g/cm^2^)	0.977 ± 0.14	0.937 ± 0.15^a^	0.926 ± 0.14^b^
Legs BMD (g/cm^2^)	1.344 ± 0.15	1.349 ± 0.14	1.328 ± 0.14^b,c^
Trunk BMD (g/cm^2^)	1.117 ± 0.11	1.086 ± 0.11^a^	1.058 ± 0.11^b,c^
Ribs BMD (g/cm^2^)	1.025r± 0.11	0.960 ± 0.09^a^	0.924 ± 0.10^b,c^
Pelvis BMD (g/cm^2^)	1.113 ± 0.13	1.105 ± 0.13^*a^	1.079 ± 0.13^b,c^
Spine BMD (g/cm^2^)	1.277 ± 0.15	1.244 ± 0.13^a^	1.220 ± 0.13^b,c^
Total BMD (g/cm^2^)	1.292 ± 0.11	1.272 ± 0.11^a^	1.254 ± 0.11^b,c^

*BMD* bone mineral density; Values are represented as mean± standard deviation and are compared by Student’s *t* test for independent samples.

^a^ – *p* <0.001 6 months vs baseline

^*a^ *p* <0.05 6 months vs baseline

^b^- *p* <0.001 12 months vs baseline

^*b^ *p* <0.05 12 months vs baseline

^c^- *p* <0.001 12 months vs 6 months

Gender-oriented analysis revealed a more significant bone loss in males at almost all measured sites (including spine—a loss of BMD of 5.67% vs 1.01% at 6 months, rising to 5.74% vs 3.16% at 12 months). Furthermore, we divided females according to menopausal status (premenopausal *N*=97, postmenopausal *N*=43). At 12 months post-LSG, men showed a significantly higher decline in total BMD than premenopausal women at all sites except the head (Table 3).

**Table 3:** Assessment of gender- and menopausal-related changes at 6 and 12 months of follow-up

% change Variable	After 6 months			After 12 months		
	PreM W	PostM W	Men	PreM W	PostM W	Men
	(*n *= 97)	(*n* = 43)	(*n* = 101)	(*n* = 97)	(*n* = 43)	(*n*=101)
General characteristics changes (%)						
BMI	28.49 ± 4.39	25.23 ± 6.03^c^	28.94 ± 5.77^a^	35.51 ± 6.27	30.44 ± 7.97^c^	34.83 ± 7.46^a^
EWL	77.28 ± 23.01	66.12 ± 17.75^c^	70.44 ± 18.19	95.32± 25.18^b^	79.40 ± 22.07^c^	84.08 ± 19.89
Bone mineral density changes (%)						
Head BMD	0.43 ± 2.39	1.45 ± 5.21	−0.22 ± 5.91	0.71 ± 2.91	1.65 ± 2.96	-0.01 ± 6.39
Arms BMD	1.77 ± 17.37	3.03 ± 15.29	3.71 ± 17.07	3.27 ± 15.96	3.32 ± 13.12	4.97 ± 16.79
Legs BMD	−1.28 ± 3.40	−0.35 ± 3.20	0.23 ± 2.94^b^	0.13 ±3.42	1.89 ± 3.53^c^	1.71 ±3.34^b^
Trunk BMD	1.40 ± 3.07	1.58 ± 3.53	4.42 ± 3.37^a,b^	3.93 ±3.87	4.06 ± 5.14	6.96 ±4.14^a,b^
Ribs BMD	4.40 ± 3.65	4.87 ± 4.65	8.46 ± 4.81^a,b^	7.52 ±5.91	7.11 ± 10.43	12.67 ±5.75^a,b^
Pelvis BMD	0.29 ± 4.19	−0.40 ± 6.62	1.19 ± 5.17	2.52 ± 4.84	2.84 ± 7.04	3.29 ± 5.32
Spine BMD	0.64 ± 5.53	1.84 ± 7.33	5.67 ± 7.45^a,b^	3.21 ±4.93	3.06 ± 5.65	5.74 ±6.09^a,b^
Total BMD	0.39 ± 3.43	1.06 ± 2.72	2.57 ± 3.49^a,b^	1.76 ±3.51	2.42 ± 3.10	4.04 ±3.27^a,b^

*PreM W* premenopausal women, *PostM W* postmenopausal women; The % change was defined as 100 X [measure 1–measure 2]/measure 1.

^a^- *p*<0.05 when comparing men- postmenopausal women

^b^ - *p*<0.05 when comparing men- premenopausal women

^c^- *p*<0.05 when comparing postmenopausal women- premenopausal women

**Fig. 1:**
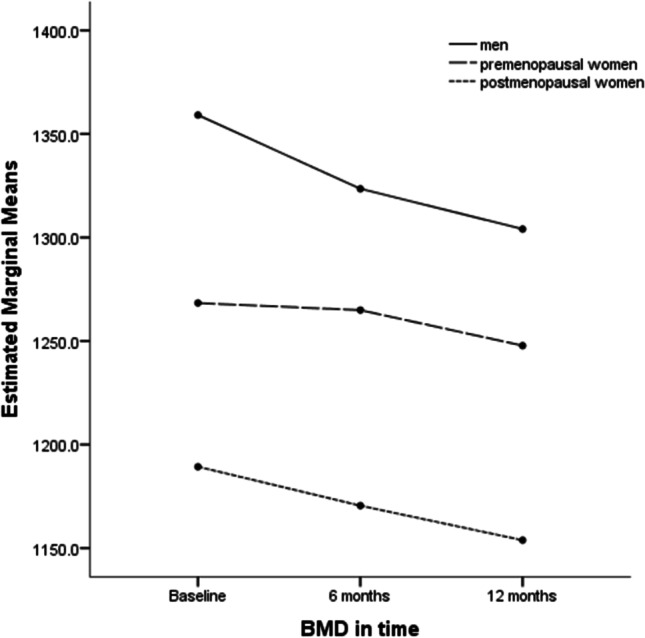
Total BMD trends over 12 months adjusted by age and BMI variation. Covariates appearing in the model are evaluated at the following values: %BMI baseline-12 months=34.32, age=41.66

To eliminate the impact of confounding factors, we adjusted the analysis for age and BMI change. GLM confirmed a greater total BMD decline in men versus premenopausal women (mean difference 2.37%, *p*<0.05). Conversely, the difference in BMD decline between men and postmenopausal women did not reach statistical significance. Analyzing total BMD trends over 12 months post-LSG using covariates model, differences in patterns of bone loss emerged. During the first 6 months post-LSG, men, premenopausal and postmenopausal women lost 2.62%, 0.27%, and 1.58% of BMD, respectively. The second period of observation (6–12 months) is similar in all three groups, revealing steady BMD decline (an additional 1.4% approximately) (Fig. 1).

## Discussion

Our study aimed to evaluate the impact of LSG on BMD and compare gender-specific trends in post-surgical bone loss. Multiple studies have demonstrated decreased DXA-evaluated BMD after bariatric surgery [21–23]. BMD decreased at almost all analyzed sites in our cohort—including spine at 6 months and continued to decline during the subsequent 6 months post-LSG. Published studies show inconsistent results regarding regional BMD evolution post-bariatric surgery. A recent study by Ieong et al., including patients who underwent either RYGB or LSG, found that, after 24 months, all subjects had a significant decrease in the femoral neck, femoral trochanter, and total hip BMD; however, only RYGB patients showed a decrease in lumbar spine BMD [16]. Conversely, a meta-analysis conducted by Tian et al. found similar regional BMD declines at all analyzed sites post-LSG and RYGB [24]. Focusing only on LSG, the meta-analysis conducted by Jaruvongvanich et al. demonstrated a significant decrease in BMD at the hip and no significant change at the lumbar spine at 6–12 months after LSG [15].

The proximal femur was almost universally found to be the most afflicted site BMD-wise post-bariatric surgery, the most plausible explanations being mechanical unloading and a potential greater susceptibility of cortical bone [21, 25–28]. Since we evaluated regional BMD through whole-body DXA scans, we could not assess proximal femur BMD but, instead, recorded legs BMD. Interestingly, legs BMD did not differ significantly from baseline at 6 months postoperatively, only showing decline 12 months post-LSG. Looking into gender-oriented analysis, we found that men did display a slight decline in legs BMD 6 months postoperatively (0.23 ± 2.94%), but the final result was driven by the apparent increase in legs BMD in women (by 1.00 ± 3.35%). This is not the first report of regional BMD increase post-bariatric surgery, but previous studies have only described lumbar spine BMD increases [29, 30]. These findings support the dual role of obesity concerning the bone. Obesity is classically regarded as a protective factor for bone health, associated with lowered fracture risk [31, 32]. However, various authors have challenged this concept, as it seems more likely that obesity has positive effects on specific skeletal sites and negatively impacts others [33–35].

One of the most consistent correlations described in the literature is between BMD decline and the extent of weight loss [14, 21, 25]. In our study, BMD deterioration correlated positively with BMI decline only at 12 months and did not correlate to EWL at 6 months and 12 months postoperatively. Conversely, Geoffroy et al., in a study including 110 patients followed-up post-bariatric surgery, found a strong correlation between BMD decline and EWL [25]. Mechanical unloading represents the simplest explanation. However, the fact that bone loss also occurs in non-weight-bearing regions and advances in research has made it increasingly clear that the etiology of post-bariatric surgery bone loss is multifactorial. This is thought to involve changes in levels of nutrients, as well as in hormonal and cytokines milieu. Proposed factors include estrogen, insulin, IGF-1, cortisol, adipokines, incretins, calcium, vitamin D and parathormone [11, 14, 24, 36, 37]. Nevertheless, connections are not straightforward since postoperative changes in some are inconsistent, while others have an unclear impact on bone health [38, 39]. Peptide YY (PYY) is a molecule that has recently gained interest in light of its role in the gut-bone axis. This anorexigenic gut hormone shows significant increase following bariatric surgery and has been proven to be a negative regulator of bone mass and strength in animal studies [40–42]. In a recent study on patients undergoing RYGB, postoperative PYY increases negatively correlated with spine BMD and bone formation marker P1NP [43]. These effects are thought to be mediated through the variety of Y-receptors (Y1-5 in humans) that modulate both feeding behavior (Y1, Y5) and bone homeostasis (Y2, Y4) [44–46].

An important finding of our study was that men displayed a more significant decline in BMD 12 months post-LSG compared with women at all sites, especially spine. This occurred although men and women displayed similar percentual BMI reductions at this point, and women had a significantly higher EWL. Therefore, simple mechanical unloading is an unlikely explanation for this difference. Instead, we hypothesize intricate contributions from the factors mentioned above may be responsible. Adipose tissue expansion leads to overexpression of aromatase, resulting in hyperestrogenism and subsequent decrease in pituitary LH, thus impairing testosterone secretion [47, 48]. Armamento-Villareal R. et al. evaluated hormonal changes in men with obesity after 12 months of lifestyle modification. They found that total and free estradiol levels drop with weight loss, but total and free testosterone levels show no improvement (possibly through a delay in LH un-suppression), and this may lead to worsening of age-related muscle and bone loss [49].

The main strength of our study, apart from including a large number of individuals, is patient distribution, which allowed valid comparisons between men, premenopausal, and postmenopausal women, respectively. This led to the key outcome, which is that 12 months post-LSG, men displayed greater BMD decline than premenopausal women, independently of weight loss and age. We could not identify a study on LSG providing a similar comparison. However, on a similarly divided cohort, the POUNDS-LOST diet trial revealed no BMD decline in men, while premenopausal women exhibited femoral neck BMD decline and postmenopausal women showed lumbar and femoral neck BMD decline [50]. Schafer et al. found that postmenopausal women had a more dramatic BMD decrease after RYGB, while BMD loss in men was less severe [51].

Our findings should be considered in the context of optimizing clinical care. If men are at greater risk for BMD loss after LSG, more careful evaluation, follow-up, and therapeutic intervention might be warranted. BMD trends over 12 months post-LSG also provide valuable information. Men seem to experience the most significant decline in BMD during the first 6 months. Therefore, this might represent the main therapeutical window for mitigating bone loss in male patients. Guideline recommendations regarding post-surgical medical and nutritional therapy are heterogeneous and non-gender oriented, but they all stress the need to ensure adequacy of calcium (1200–1500mg/day), vitamin D (3000UI/day, with a goal of >30ng/mL), protein (a minimal of 60 g/day and up to 1.5 g/kg ideal body weight per day), and physical exercise [52–54]. Since the loss of lean mass has been shown to positively correlate with the bone loss [55, 56], its preservation might also represent an intervention target. There is currently insufficient evidence to outline an optimal exercise regime to promote bone health. Generally, a combination of aerobic (preferably weight-bearing—e.g., walking, for a minimum of 150 min, with a goal of 300 min/week), strength training (2–3 times/week), and flexibility exercises is recommended [52, 53, 57]. Although swimming is classically regarded as less effective [58], recent animal studies reveal beneficial skeletal outcomes [59–62]. This warrants future consideration, especially for the immediate perioperative setting when patients are still overweight or obese and might benefit from exercises that are less strenuous on articulations.

A limitation of this study is its short duration. However, evidence from longer-term research on bariatric surgery suggests the outcome does not improve with time, although weight loss mostly ceases after 12 months. Villarasa et al. revealed that postoperative decreases in BMD continue up to 3 years [56], while more recent studies have confirmed continued decline, albeit at a slower rate, up to 5 [63, 64]–7 [65] years postoperatively.

Another limitation is the fact that we solely relied on DXA scans to assess bone density. Multiple studies have shown that the overlying fat can produce artifactual increases. They suggest quantitative computed tomography (QCT) as a more appropriate tool for evaluating bone mass in individuals with obesity, especially in the context of significant weight and body composition changes [66–68]. However, QCT is an expensive tool, unlikely to be implemented into routine clinical practice in the foreseeable future and DXA scans remain the mainstay of bone evaluation. Furthermore, Yu et al. conclude that, while on an individual level it might be challenging to interpret DXA evaluated changes in BMD with profound concomitant changes in body composition, group results are bound to be reliable [68].

## Conclusion

Our study adds to the body of evidence that LSG negatively impacts BMD, stressing that bone health should represent one of the priorities of postoperative care. Moreover, the possibility that men suffer more profound bone loss after LSG warrants exploration and confirmation on larger cohorts since it is bound to modulate clinical practice.
